# Cost-effectiveness analysis of dapagliflozin in the management of heart failure with reduced ejection fraction (HFrEF): a systematic review

**DOI:** 10.1186/s12962-022-00396-7

**Published:** 2022-12-01

**Authors:** Ghader Mohammadnezhad, Behniya Azadmehr, Mehdi Mirheidari, Nazila Yousefi

**Affiliations:** 1 Student Research Committee, School of Pharmacy, Shahid Beheshti University of Medical Sciences, Tehran, Iran; 2 Department of Pharmacoeconomics and Pharma Management, School of Pharmacy, Shahid Beheshti University of Medical Sciences, Tehran, Iran

**Keywords:** Dapagliflozin, SGLT-2 inhibitors, Economic evaluation, Cost-effectiveness analysis, Heart failure

## Abstract

**Objectives:**

This study was aimed to systematically review published economic studies to determine whether dapagliflozin, a sodium-glucose co-transporter inhibitor, plus standard care therapy (SCT) is cost-effective in heart failure with reduced ejection fraction (HFrEF).

**Method:**

We searched relevant keywords in PubMed, Scopus, Web of science, and Google Scholar to find related articles. Costs, QALYs, ICERs were extracted from eligible studies.

**Results:**

Ten studies finally included in the systematic review. The results of quality assessment of the study showed that a reasonable quality of all studies. Incremental QALYs were in favor of dapagliflozin plus SCT treatment regimen. In all the studies, the incremental costs per QALY was below the willingness-to-pay (WTP) threshold with the exception of one study in United Kingdom which the ICER and WTP were $83,650 and $50,000. All the studies determined the National Health Care perspective. The highest and lowest ICERs were $83,650 and $1991 per QALY in United Kingdom and Thailand, respectively.

**Conclusion:**

Results of cost-effectiveness analyses showed that adjunct dapagliflozin plus SCT is cost-effective compared to SCT alone despite the additional costs of the drug. Finally it can be concluded that dapagliflozin is a worldwide cost-effective as an adjunct medicine in HFrEF management.

**Supplementary Information:**

The online version contains supplementary material available at 10.1186/s12962-022-00396-7.

## Introduction

Heart failure is a chronic disease associated with impaired blood flow to or from the heart. Heart failure with reduced ejection fraction (HFrEF) occurs when the left ventricular stroke volume falls below 40%, which can lead to cardiac remodeling [1–5]. HFrEF is a major public health concern and is associated with significant morbidity and mortality. There have been significant scientific breakthroughs in the treatment of HFrEF in recent decades, and the ability to treat the disease has never been better [6, 7]. Despite all these breakthroughs, the morbidity and mortality of the disease are still high, with a 5-year survival rate of 25% of hospitalizations. In addition, heart failure is associated with symptoms such as dyspnea, peripheral edema, and fatigue. Based on the results of a 5-year study of 18,398 patients, the mortality rate for HFrEF was estimated to be 75%. These results suggest that current pharmacotherapies and surgical interventions have poor survival rates [5, 8–10].

Sodium-Glucose co-Transporter 2 inhibitors (SGLT-2i) are a recent development in cardiovascular therapy, which significantly improves the prognosis of HFrEF. SGLT-2i such as empagliflozin, dapagliflozin, and canagliflozin, which are approved as antidiabetic agents, have been attributed cardioprotective and promising nephroprotective effects beyond glycemic control, especially with the clinical results of dapagliflozin [1, 11, 12]. In an 18-month clinical trial involving 4744 participants, dapagliflozin 10 mg/day showed a significant therapeutic effect in HFrEF compared with placebo. Although the efficacy of dapagliflozin as an adjunct to heart failure standard therapy has been established, it seems necessary to monitor the economic impact and investigate whether or not the addition of dapagliflozin to standard therapy is economically sound [13, 14].

Due to limited financial resources, and high costs of HFrEF treatment with dapagliflozin, healthcare systems are simultaneously looking for the best efficacy and the lowest cost. Therefore, cost-effectiveness analysis should be performed to select an appropriate treatment strategy by pharmaceutical decision makers. Against this background, this study aimed to systematically assess the cost-effectiveness of dapagliflozin as add-on therapy to standard care therapy (SCT) in patients with HFrEF [15].

## Research question

This systematic review was conducted following the Preferred Reporting Items for Systematic Reviews and Meta-Analyses (PRISMA) to answer the question, “Is the addition of dapagliflozin 10 mg/day to SCT cost-effective compared to SCT alone in HFrEF patients?”.

## Method

### Search strategy

To find relevant articles, we searched online databases for published articles on November 15, 2021, including PubMed, Scopus, Web of Science, Cochrane Library, and ScienceDirect. We used a combination of the following keywords: “dapagliflozin,” “cost-effectiveness,” “cost–benefit,” “cost-minimization,” “pharmacoeconomic study,” “economic study,” “pharmacoeconomic evaluation,” “economic evaluation,” and “heart failure.” Table 1 provides the exact searches in all databases and search engines.

**Table 1:** Primary Characteristics of the Studies

Study (year)	Country	Perspective	Currency/year	Economic study type	Time horizon	Discount rate
Paritzo et ,al. 2021	UK	Healthcare Payer	US Dollars/2020	Markov model	Life time	3%
Krittayaphong et al., 2020	Thailand	Healthcare System	Thai Baht/2020	Markov model	Life time	3%
Mendoza et al., 2021	Philippines	Public Healthcare provider	Philippine Peso/2021	Markov model	Life time	3%
Savira et al., 2020	Australia	Australian Healthcare	Australian Dollars /2019	Markov model	Life time	5%
Isaza et al., 2021	US	US Healthcare Sector	US Dollars/2020	Markov model	Life time	3%
McEwan et al., 2020	UK, Germany, and Spain	Euro Multi-national Healthcare System	British Pound/2019	Markov model	Life time	Spain and Germany: 3%
			Euro/2019			UK: 3.5%
Yao et al., 2020	China	Chinese Healthcare Payers	Chinese Renminbi/2020	Markov model	15 years	4.2%
Liao et al., 2021	South Korea, Australia, Taiwan, Japan, and Singapore	Asia–Pacific Region Healthcare Systems	US Dollars/2020	Markov model	18 months	3%
					30 years	
Gil-Rojas, 2021	Colombia	Colombian Health System	US Dollars/2020	Markov model	5 years in the base case	5%
					10 years in sensitivity analyses	
					16 years for the life expectancy analysis	
Jiang et al., 2021	China	Chinese Medical and Health System	US Dollars/2021	Markov model	5 years	5%
					10 years	
					15 years	
					20 years	

### Eligibility criteria

Eligible studies were: (1) Economic analyses including cost-effectiveness analyses, cost-utility analyses, cost–benefit analyses, and cost-minimization analyses); (2) Studies consisting of dapagliflozin plus SCT versus SCT alone; and (3) Studies with the HFrEF population over 18 years of age. We defined a PICO to organize the systematic review. Population of the study was the patients with HFrEF, intervention was adjunct dapagliflozin, comparator was SCT or adjunct empagliflozin, and outcomes were costs, QALYs, and ICER.

All search results were reviewed for inclusion criteria by two independent investigators (MM and BA). Any conflict was resolved after a discussion between all researchers. Full texts were selected based on eligibility criteria. Conference proceedings, abstracts, and articles without full-text access were excluded. For better comparison and more accurate judgment, all costs were converted to US dollars, and the conversion method was based on the value of the currency used in the study to US dollars at the time of the study.

### Quality assessment

The Consolidated Health Economic Evaluation Reporting Standards (CHEERS) checklist was applied for the quality assessment of the included studies. CHEERS checklist contains 24 items about different sections of economic studies. Studies that scored more than 75% were considered as high quality.

## Results

### Database search findings

**Fig. 1:**
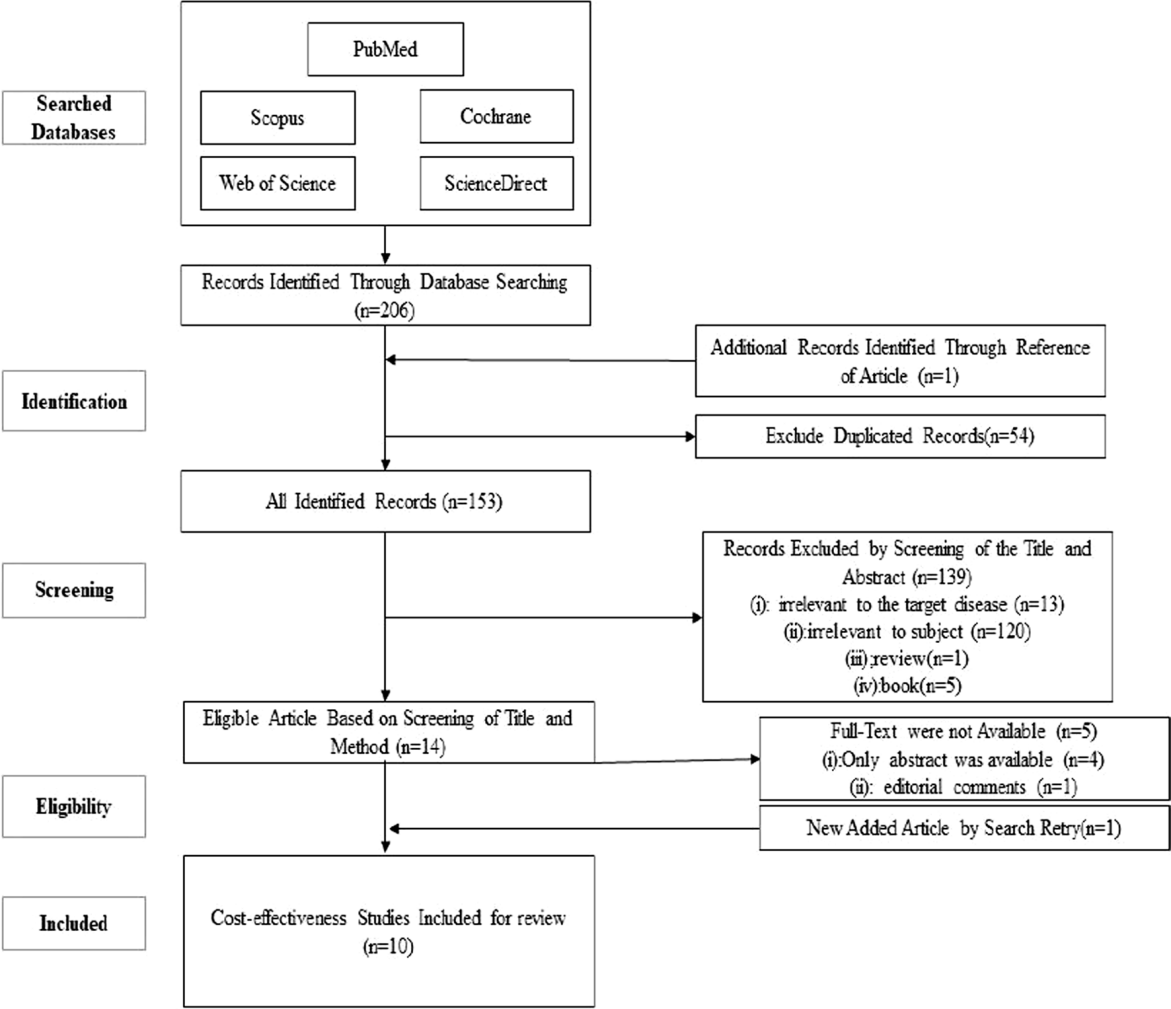
PRISMA flow diagram

In this study, we used the PRISMA flow diagram to include all relevant studies that met the eligibility criteria (Fig. 1). The primary search retrieved 206 articles by searching the databases according to the search query. After removing duplicates, 153 articles remained. Then, 139 articles were removed by reviewing the title and abstract, and 14 studies remained to be reviewed by full-text. Finally, ten economic evaluations remained and others were deleted for not meeting the inclusion criteria.

### Overview of the studies

Finally, 10 studies were included and subjected to extraction of study characteristics and economic data. All economic evaluations were cost-utility analyses and were based on a Markov model. The studies included economic evaluations in developing and developed countries, including Thailand, the Philippines, Colombia, China, Australia, the United States (US), South Korea, Taiwan, Japan, the United Kingdom (UK), Germany, and Spain. All studies had been published in 2020 and 2021, which shows how trendy the topic was [16–25]. The time horizon of five studies was life time, one study had a time horizon of 15 years [23], and the other had a time horizon of 5 years [24]. Some studies included two [20] or more different time horizons. The discount rates of the included studies were 3% in six studies, and > 3% discount rate (Table 1) was used in four others [18, 22–24]. A Markov model was used in all studies, and the national healthcare system perspective was included in the costing in all studies. The population of all studies included patients with HFrEF and an age group older than 18 years. Two studies reported > 18 years, whereas the other studies did not report age [17, 20]. Four studies excluded patients with a glomerular filtration rate < 30 mL/min/1.73m^2^ [17, 18, 20, 22]. The characteristics of the included articles are summarized in Table 1.

### Quality assessment

The CHEERS checklist was used to assess the quality of the studies and presented in Table 2. The results showed slightly different quality scores, but all showed an acceptable level of quality (more than 80% of the total score). Based on this checklist, the study received a score of one if it fully and transparently addressed the intended question. A score of 0.5 was assigned if it was only partially mentioned, and a score of zero was assigned if the study did not cover the question. Finally, the average score of the studies was 94.6%, showing the high-quality level of the included studies. All studies had made a point of referring to their purpose and nature in their title (Question 1). All studies had included a full abstract with purpose, perspective, method, result, and conclusion, except Gil-Rojas et al. [24], which did not include a full abstract (Question 2). All studies had addressed the background of the topic and stated the study question and its relevance to the topic (Question 3). All studies had provided the characteristics of the base-case population, setting, location, and perspective (Question 4–6). All studies had provided a good description of the comparators except that of Mendoza et al. [19] who did incompletely (Question 7). All studies had clearly stated the time horizon over which costs and consequences were assessed (Question 8). All studies had explicitly stated the discount rate, except that of Isaza et al. [21] (Question 9). All studies, except that of Jiang et al. [16], had provided an explicit description of the outcomes, design features (if single study-based estimates were used) or methods to include appropriate studies (if synthesis-based estimates were used), study target population and methods, and study approaches to estimate resources and costs (Question 10–13). Eight studies had reported data on estimated unit costs and the method used to convert costs to a common currency and two studies reported it partially [18, 21] (Question 14). All studies had fully discussed the reason for choosing a particular decision model (Question 15). Three studies had not addressed all of the assumptions used in the analytical decision model [18, 21, 25] (Question 16). All studies had described the analytical method of evaluation in detail (Question 17). All studies had met the standard in reporting study parameters such as values, ranges, and references (Question 18). All studies had fully reported the ICERs and economic outcomes of the targeted intervention (Question 19). With the exception of a study by Mendoza et al. [19] (Question 20), all studies had successfully and comprehensively described uncertainty. Except for the Chinese studies, all studies had characterized heterogeneity by reporting differences in costs and outcomes [16, 23] (Question 21). All studies had mentioned key findings, generalizability, limitations, and current knowledge (Question 22). All studies, except those of Krittayaphong et al. and Jiang et al. [16, 25], had reported their funding source and their sponsor's role in the study (Question 23). All studies had clarified their conflicts of interest when possible based on the circumstances of the contributors (Question 24).

**Table 2:** CHEERS Checklist

Items/studies	Title	Abstract	Background and objectives	Target population and subgroups	Setting and location	Study perspective	Comparators	Time horizon	Discount rate	Choice of health outcomes	Measurement of effectiveness
Gil-Rojas [24]	⬆	⬇	⬆	⬆	⬆	⬆	⬆	⬆	⬆	⬆	⬆
Liao [20]	⬆	⬆	⬆	⬆	⬆	⬆	⬆	⬆	⬆	⬆	⬆
Yao [23]	⬆	⬆	⬆	⬆	⬆	⬆	⬆	⬆	⬆	⬆	⬆
Mc Ewan [18]	⬆	⬆	⬆	⬆	⬆	⬆	⬆	⬆	⬆	⬆	⬆
Isaza [21]	⬆	⬆	⬆	⬆	⬆	⬆	⬆	⬆	⬇	⬆	⬆
Parizo [17]	⬆	⬆	⬆	⬆	⬆	⬆	⬆	⬆	⬆	⬆	⬆
Savira [22]	⬆	⬆	⬆	⬆	⬆	⬆	⬆	⬆	⬆	⬆	⬆
Mendoza [19]	⬆	⬆	⬆	⬆	⬆	⬆	⬅	⬆	⬆	⬆	⬆
Krittayaphong [25]	⬆	⬆	⬆	⬆	⬆	⬆	⬆	⬆	⬆	⬆	⬆
Jiang [16]	⬆	⬆	⬆	⬆	⬆	⬆	⬆	⬆	⬆	⬆	⬆

Items/studies	Measurement and valuation of preference based outcomes	Estimating resources and costs	Currency, price date, and conversion	Choice of model	Assumptions	Analytical methods	Study parameters	Incremental costs and outcomes	Characterizing uncertainty	Characterizing heterogeneity	Study findings, limitations, generalizability, and current knowledge	Source of funding	Conflict of interests	Total
Gil-Rojas [24]	⬆	⬆	⬆	⬆	⬆	⬆	⬆	⬆	⬆	⬆	⬆	⬆	⬆	23.0
Liao [20]	⬆	⬆	⬆	⬆	⬆	⬆	⬆	⬆	⬆	⬆	⬆	⬆	⬆	24.0
Yao [23]	⬆	⬆	⬆	⬆	⬆	⬆	⬆	⬆	⬆	⬅	⬆	⬆	⬆	23.5
Mc Ewan [18]	⬆	⬆	⬅	⬆	⬇	⬆	⬆	⬆	⬆	⬆	⬆	⬆	⬆	22.5
Isaza [21]	⬆	⬆	⬅	⬆	⬇	⬆	⬆	⬆	⬆	⬆	⬆	⬆	⬆	21.5
Parizo [17]	⬆	⬆	⬆	⬆	⬆	⬆	⬆	⬆	⬆	⬆	⬆	⬆	⬆	24.0
Savira [22]	⬆	⬆	⬆	⬆	⬆	⬆	⬆	⬆	⬆	⬆	⬆	⬆	⬆	24.0
Mendoza [19]	⬆	⬆	⬇	⬆	⬆	⬆	⬆	⬆	⬅	⬆	⬆	⬆	⬆	22.0
Krittayaphong [25]	⬆	⬆	⬆	⬆	⬇	⬆	⬆	⬆	⬆	⬆	⬆	⬇	⬆	22.0
Jiang [16]	⬅	⬇	⬆	⬆	⬆	⬆	⬆	⬆	⬆	⬇	⬆	⬇	⬆	20.5

In all trials, dapagliflozin plus SCT for the treatment of HFrEF was the intervention arm, and SCT or SCT plus placebo or SCT plus empagliflozin was the comparator. The trials captured drug costs, iatrogenic adverse events (AEs), HF-related morbidity and mortality, and healthcare services. Total QALYs and total costs had been reported in all studies, except that of Mendoza et al. [19]. However, QALYs varied across countries. In addition, Isaza et al. had presented QALYs for diabetic and non-diabetic patients separately [21]. Paritzo et al. had also calculated different QALYs according to the severity of the disease, and Liao et al. had reported different QALYs based on the four different scenarios [17, 20]. The highest and lowest utility were found in Asia–Pacific countries (South Korea, Australia, Japan, and Singapore) [20]. All studies had reported ICERs that were below the willingness-to-pay (WTP) thresholds in their study context, except for a study by Parizo et al. in England [26]. Thus, dapagliflozin was cost-effective as add-on therapy vis-a-vis SCT alone in most of the economic analyses. Details been demonstrated in Table 3.

**Table 3:** Outcomes and Main Measures of the Studies

Comparator	Types of cost included	Cost	Effectiveness	ICER (USD/QALY)	WTP (per QALY)	Ref**.**
SCT	Medication costs, add-on dapagliflozin, HF hospitalizations, and urgent visit HF costs	Incremental costs of dapagliflozin vs. SCT:	Dapagliflozin plus SCT:	Dapagliflozin plus SCT vs SCT:	$100,000	[21]
		1. All patients: $42,800	1. All patients: 5.36	1. All patients: $68,300		
		2. No diabetes:$40,100	2. No diabetes: 5.86	2. No diabetes: $69,600		
		3. Diabetes: $46,500	3. Diabetes: 4.66	3. Diabetes: $66,800		
			SCT alone:			
			1. All patients: 4.73			
			2. No diabetes: 5.28			
			3. Diabetes: 3.96			
SCT	Medication costs, treatment, monitoring, adverse events, worsening HF events, and CV death	-UK:	-UK:	Dapagliflozin plus SCT vs SCT only:	UK: £20000 ,Germany and Spain : €20000 in	[18]
		1. Dapagliflozin plus SCT: $21821.9	1. Dapagliflozin plus SCT: 4.61	UK: $7743		
		2. SCT only: $18,124.6	2. SCT only:4.13	Germany: $6143		
		-Germany:	-Germany:	Spain: $10742		
		1. Dapagliflozin plus SCT: $28925.4	1. Dapagliflozin plus SCT:4.72			
		2. SCT only: $25,863.6	2. SCT only:4.22			
		- Spain:	-Spain:			
		1. Dapagliflozin plus SCT: $27,785.7	1. Dapagliflozin plus SCT:4.72			
		2. SCT only: $22,431.8	2. SCT only:4.22			
SCT	Hospitalization, prescription medications for HF, HF-associated readmissions in hospitals and specialist visit fees	Model 1.	Model 1.	Model 1.	$8573.4	[23]
		1. Dapagliflozin plus SCT: $5,829.4	1. Dapagliflozin plus SCT: 4.82	18-month time horizon: $14,883		
		2. SCT only: $4,377.1	2. SCT only:4.44	15-year time horizon: $3827.6		
		Model 2.	Model 2.	Model 2.		
		1. Dapagliflozin plus SCT: $5,858.4	1. Dapagliflozin plus SCT: 4.82	Dapagliflozin plus standard care vs. standard care only:		
		2. SCT only: $4,436.6	2. Standard care only: 4.44	$3732.3		
SCT	1. Direct: Healthcare services and medications, 2. Indirect: loss of productivity caused by morbidity and mortality	-Costs in different time horizons:	-In different time horizons:	Add-on dapagliflozin vs. SCT alone: Korea: $5277	$25,000 and $75,000	[20]
		(a) 18 months:	(a) 18 months:	Australia: $9980		
		1. Dapagliflozin plus SCT: $16,719	1. Dapagliflozin plus SCT:2.10	Japan: $16,705		
		2. SCT: $15,732	2. SCT: 2.08	Singapore: $23,227		
		(b)30 years:	(b)30 years:	Taiwan:		
		1. Dapagliflozin plus SCT: $104,632	1. Dapagliflozin plus SCT: 13.14	-Based ICER: $12,305		
		2. SCT: $87,940	2. SCT: 11.6	-Different Time Horizons:		
		-Costs in different discount rates:	-In different discount rates:	(a) 30 years: $10,832		
		(a) 0%:	(a) 0%:	(b) 18 months: $37,386		
		1. Dapagliflozin plus SCT: $104,288	1. Dapagliflozin plus SCT:13.1	-Different discounting rates:		
		2. SCT: $90,112	2. SCT:11.89	(a) 0%: $11,681		
		(b)10%:	(b)10%:	(b) 10%: $13,007		
		1. Dapagliflozin plus SCT: $62,215	1. Dapagliflozin plus SCT:7.82			
		2. SCT: $55,130	2. SCT:7.25			
SCT	Hospitalization, medicines, and treatment of adverse events	1. Dapagliflozin plus SCT: $1747.8	1. Dapagliflozin plus standard therapy: 6.92	Dapagliflozin plus standard care vs standard care only: $1991 per QALY	$5131	[25]
		2. Standard care only: $559.3	2. Standard care only:6.33			
SCT	Costs of medicines, HF hospitalization, and adverse event management	N.R	N.R	Dapagliflozin plus standard therapy vs standard therapy:	180,500 PHP	[19]
				- All patients:		
				(A) PHP40: $3108		
				(B) PHP44: $3,6380		
				(C) PHP46.5: $3,638		
				-Diabetic:		
				(A) PHP40: $2,708		
				(B) PHP44: $2,560		
				(C) PHP46.5: $2,321		
				-Non-diabetic:		
				(A) PHP40: $5698		
				(B) PHP44: $5372		
				(C) PHP46.5: $4852		
SCT	Cost of dapagliflozin and SCT, HF hospitalization, and death	1. Dapagliflozin plus standard therapy: 19,627.8 AUD	1. Dapagliflozin SCT: 2.8	Dapagliflozin plus standard care vs standard care only: 8,861 AUD	50,000 AUD	[22]
		2. Standard care only: 17,079.6 AUD	2. SCT only: 2.5			
SCT	Dispensing fees, the drug plan payment, and the beneficiary copayment	1. Dapagliflozin:	1. Dapagliflozin:	Dapagliflozin vs standard of care: total $83650	$100,000 and $150,000	[17]
		(A) Mild impairment: $202,646	(A) Mild impairment:6.7	(A) Mild: $78,483		
		(B) Moderate impairment: $172,045	(B) Moderate impairment: 4.2	(B) Moderate: $97,608		
		2. SCT:	2. SCT:			
		(A) Mild impairment:$157,833	(A) Mild impairment: 6.1			
		(B) Moderate impairment: $141,783	(B) Moderate impairment: 3.9			
SCT	Medicines, follow-up, clinical events by the AEs of the medicines or morbidity of the disease (HF care, emergency medical consultation, amputation, diabetic ketosis)	-All patients:	-All patients:	SCT plus dapagliflozin vs. SCT plus Placebo:	Variable	[24]
		1. Dapagliflozin plus standard therapy: $4,611.2	1. Dapagliflozin plus standard therapy: 2.689	-All patients		
		2. Standard care plus placebo: $3,808.3	2. SCT plus placebo: 2.554	$5,946		
		-Diabetic patients:	-Diabetic patients:	-Diabetic patients:		
		1- Dapagliflozin plus standard therapy: $5,476	1- Dapagliflozin plus standard therapy: 3.475	$4,881.2		
		2- Standard care plus placebo: $4,767	2- SCT plus placebo: 3.313	-Non-diabetic patients:		
		-Non-diabetic patients:	-Non-diabetic patients	$6,867.5		
		1. Dapagliflozin plus standard therapy: $3,926	1. Dapagliflozin plus standard therapy: 3.734			
		2. Standard care plus placebo: $3,054.5	2. SCT plus placebo: 3.603			
SCT alone and in combination with empagliflozin	The standard treatment cost included ACEI, ARBs,	Dapagliflozin group: $4,870.68	1. Dapagliflozin plus SCT: 3.87	$5,541 in the dapagliflozin strategy and $6,946 in the empagliflozin strategy.	$11,008	[16]
	Beta-blockers, spironolactone, and diuretic. The cost of hospitalization for HF was from the China	Empagliflozin group: $5,021.93	2. SCT alone (control 1): 3.64			
	Health Statistics Yearbook 2020, which included town-level,	Control 1 group from DAPA-HF trial: $3,596.25	1. Empagliflozin plus SCT: 3.66			
	County-level, municipal, provincial, and ministerial hospitals.	Control 2 group from EMPEROR-Reduced: $4,118.86	2. SCT alone (control 2): 3.53			

^a^*SCT* standard care therapy, *ICER* incremental cost-effectiveness ratio, *QALY* quality-adjusted life-years, USD United States dollars, *HF* heart failure, *CV* cardiovascular, *N.R*: not reported, *PHP* Philippine peso, *AUD* Australian dollars, *AE* adverse events, *ACEI* angiotensin-converting enzyme inhibitors, *ARB* angiotensin receptor blockers, *WTP* willingness to pay threshold

In a cost-utility analysis, conducted in Europe by McEwan et al. [18], the countries studied were the UK, Germany, and Spain. QALYs for the addition of dapagliflozin to SCT were 4.61 compared with SCT alone, which was 4.13 in the UK. In Germany and Spain, QALYs were 4.72 and 4.22, respectively. The total cost of the additional treatment with dapagliflozin plus SCT was £16,408 compared with SCT alone, which was £13,628, in the UK in Germany, the total cost was €25,328 versus €22,647. In Spain, the total cost was €24,330 compared to €19,642. ICERs in the UK, Germany, and Spain were €5822, €5379, and €9406, respectively. Finally, the PSA shows that dapagliflozin was cost-effective in 96% of the cases. An addition of dapagliflozin by 5.7% resulted in a 5-year increase in survival probability.

Parizo et al. [17] conducted an economic analysis of dapagliflozin in the UK NHS cardiovascular setting in 2021. There were two different levels for which QALYs, costs, and ICERs were estimated, including mild and moderate HFrEF. The level of QALYs as add-on therapy with dapagliflozin and SCT in mild failure was 6.7 versus 6.1 and in moderate failure was 4.2 versus 3.9. The total cost of add-on treatment with dapagliflozin and SCT in mild failure was $202,646 versus $157,833. And in moderate failure, it was $172,045 versus $141,783. ICERs in mild and moderate failure were $78,483 and $97,608 per QALY, respectively. Dapagliflozin is a cost-effective add-on from a payer perspective in the UK in HFrEF patients.

In 2021, Isaza et al. [21] conducted a cost-utility analysis based on the lifetime horizon Markov model from a US healthcare perspective, they used dapagliflozin as an add-on therapy to guideline-directed medical therapy (GDMT). QALYs, incremental costs, and ICERs were calculated for all, diabetic, and non-diabetic patients for GDMT plus dapagliflozin versus GDMT alone. In the GDMT plus dapagliflozin group, QALYs were 5.36, 5.86, and 4.66 for all patients, non-diabetic, and diabetic patients, respectively, and in the GDMT group, QALYs were 4.73, 5.28, and 3.96, respectively. The incremental costs of dapagliflozin compared with GDMT were $42,800, $40,100, and $46,500 in all patients, non-diabetic, and diabetic patients, respectively. The estimated ICERs for dapagliflozin plus GDMT compared with GDMT alone were $68,300, $69,600, and $66,800 per QALY, respectively. Finally, adding dapagliflozin to GDMT was cost-effective in 94% (84% in non-diabetics and 95% in diabetics) of 10,000 case simulations. Subgroup analyses showed that ICERs differed little between two subgroups (without diabetes: $69,600, with diabetes: $66,800 per QALY gained).

In a cost-utility analysis developed by Krittayaphong et al. [25] in 2020, the QALYs of dapagliflozin as add-on therapy and SCT were 6.92 versus 6.33. The cost was ฿ (Thai Baht) 54,405 versus 17,442 from the healthcare system perspective. The estimated ICER was ฿62,090 per QALY (1991 $/QALY). The ICER was lower than the study's WTP threshold (5131 $/QALY). At a WTP of THB 120,000/QALY, dapagliflozin compared to standard treatment had a 0.87 probability of being cost-effective.

In another economic evaluation, to evaluate the cost-utility of dapagliflozin, Yao and colleagues [23] designed a Markov model with a 15-year time horizon from the perspective of a Chinese healthcare payer. The study was done in two different models, in model 1 the stages if HFrEF was based on NYHA in model 2 was based on hospitalization and non-hospitalization. The total costs for the dapagliflozin plus SCT regimen were $5829.4 and for SCT $4377.1 in model 1 and respectively, $5858.4 and $4436.6. In both the models, QALY in dapagliflozin plus SCT regimen was 4.82, in SCT alone was 4.44. Based on the results of this study, we concluded that dapagliflozin is a markedly effective option as add-on therapy in China.

Gil-Rojas et al. [24] evaluated the cost-effectiveness of their study from the perspective of the Colombian health system. The time horizon of the study was 5 years. QALYs, costs, and ICERs were estimated in all, diabetic, and non-diabetic patients for dapagliflozin plus SCT versus SCT plus placebo. In the dapagliflozin plus SCT group, QALYs were 2.689, 3.734, and 3.475 in all, non-diabetic, and diabetic patients, respectively, and in the SCT plus placebo group, QALYs were 2.554, 3.603, and 3.313 in all, non-diabetic, and diabetic patients, respectively. The costs of dapagliflozin plus SCT were $4611.2, $3926, and $5476 in all, non-diabetic, and diabetic patients, respectively. The costs for SCT plus placebo were $3808.3, $3054.5, and $4767 for all, non-diabetic, and diabetic patients, respectively. The estimated ICERs for dapagliflozin plus SCT versus SCT plus placebo were $5946, $6867.5, and $4881.2 per QALY in the three subgroups mentioned. In 97% of the analyses, the simulations resulted in an ICER below the WTP threshold.

In 2021, Liao et al. [20] conducted another cost-utility analysis to determine the cost-effectiveness of dapagliflozin plus SCT compared with SCT in Korea, Australia, Taiwan, Japan, and Singapore. Two different time horizons of 18 months and 30 years and two different discount rates of 0% and 10% were considered to estimate QALYs and costs. The perspective of the study was the healthcare systems of the Asia–Pacific region. A Markov model was developed. At an 18-month time horizon, QALYs were 2.10 and 2.08 for dapagliflozin plus SCT and SCT alone, respectively, and at a 30-year time horizon, QALYs were 13.14 and 11.6 for dapagliflozin plus SCT and SCT alone, respectively. At a discount rate of 0%, QALYs were 13.1 and 11.89 for dapagliflozin plus SCT and SCT alone, and at a discount rate of 10%, QALYs were 7.82 and 7.25 for dapagliflozin plus SCT and SCT alone. At an 18-month time horizon, costs were $16,719 for dapagliflozin plus SCT and $15,732 for SCT alone, and at a 30-year time horizon, costs were $104,632 for dapagliflozin plus SCT and $87,940 for SCT alone. At a discount rate of 0%, the costs were $104,288 and $90,112 for dapagliflozin plus SCT and SCT alone, respectively, and at a discount rate of 10%, the costs were $62,215 and $55,130 for dapagliflozin plus SCT and SCT alone, respectively. The estimated ICERs at0% and 10% discount rates were $11,681 and $13,007 per QALY, and at the 18-month and 30-year time horizons were $37,386 and $10,832 per QALY.

In 2021, Mendoza et al. [19] conducted a cost-utility model in the Philippines that aimed to determine the cost–benefit of dapagliflozin as add-on therapy in HFrEF from the perspective of public health providers. The time horizon of the study was life time. QALYs and total costs were not mentioned in this study, but the ICERs for different drugs with costs of PHP40, PHP44, and PHP46.5 were PHP160,983, PHP177,986, and PHP188,450, respectively. Although the ICER was below the WTP threshold, dapagliflozin was practically not cost-effective in the Philippines due to out-of-pocket expenses and different medical reimbursement regulations in the country. In the simulation study, dapagliflozin was cost-effective as an add-on to SCT for HFrEF patients in the Philippines, especially in diabetic patients. The addition of dapagliflozin to SCT was 58% and 64% cost-effective for HFrEF patients when the unit cost of the drug was PHP44.00 and PHP40.00, respectively. For diabetic patients with HFrEF, it was 72% and 76% cost-effective when the unit cost was similar. Savira et al. evaluated the cost-effectiveness of dapagliflozin as add-on therapy to standard treatment compared with current standard therapy. The total cost of treatment from an Australian healthcare perspective was $24,753,415 with SCT and $28,455,855 with SCT plus dapagliflozin, with QALYs of 2.5 for SCT and 2.8 for SCT plus dapagliflozin. The ICER for the study was $12,842 per QALY. The ICER was below the study threshold. Dapagliflozin was 98.8% cost-effective in Australia.

Jiang and coworkers [16] evaluated the cost-effectiveness of dapagliflozin or empagliflozin in addition to SCT of HFrEF in China. Add-on therapy with dapagliflozin was more effective (3.87 QALYs versus 3.64 QALYs) and dapagliflozin had greater efficacy in HFrEF patients (0.23 and 0.13 incremental QALYs for dapagliflozin and empagliflozin, respectively, compared with SCT alone). Although the addition of SGLT-2i was associated with higher costs for patients with heart disease, this intervention extremely improved health-related quality of life, cost of care, and cardiovascular death. The economic analysis confirmed these claims. ICERs were $5541.00 per QALY for the dapagliflozin strategy and $6946.69 per QALY for the empagliflozin treatment strategy. Deterministic sensitivity analysis showed that cardiovascular death was the most important determinant of economic evaluation rather than the SGLT-2i cost. According to the PSA indicated in the WTP of $11,008 per QALY, the probability of add-on dapagliflozin and empagliflozin being cost-effective was 70.5% and 55.2%, respectively.

In all studies, dapagliflozin plus SCT was the intervention, and SCT (in some studies plus placebo or empagliflozin) was the comparator. All studies recorded drug costs, drug-related adverse events, HF-related morbidity and mortality, and healthcare services. Except for a study by Mendoza et al., all studies reported QALYs and total costs, although QALYs varied across countries. In addition, Isaza et al. calculated QALYs separately for diabetic and non-diabetic patients [21]. Paritzo et al. calculated different QALYs based on disease severity, and Liao et al. reported QALYs based on four different scenarios [17, 20]. The highest and lowest QALYs were found in the Asia–Pacific region (Korea, Australia, Japan, and Singapore) [20]. In all studies, the reported ICERs were below WTP thresholds, and dapagliflozin was cost-effective as add-on therapy in HFrEF. In all studies, a tornado diagram was presented to describe the sensitivity of an issue to changes in the assumed variables. In most studies, the cost of the variables of SCT alone (or with placebo), cost of SCT plus dapagliflozin, and hazard ratio of cardiovascular hospitalization or mortality were the factors with the greatest impact on the results of the analyses.

## Discussion

To our knowledge, this article is the most complete systematic review conducted to investigate the cost-effectiveness of an SGLT-2i, dapagliflozin, as an add-on option plus SCT in the treatment of HFrEF. In all included studies, the ICER had been compared with the national WTP of the target country. In all studies, SCT had been selected as the comparison arm. SCT includes all diagnostic, nursing, and administrative interventions according to the HFrEF guidelines. Costs incurred at SCT include specialist visits, medications, hospitalizations, and management of adverse events [5, 27, 28]. Our objective was to assess the cost-effectiveness of all reported trials in the adopted area of PICOTS, compare the costs and consequences of different treatment guidelines, focus on the benefits of adding dapagliflozin, and examine sensitivity analyses.

**Fig. 2:**
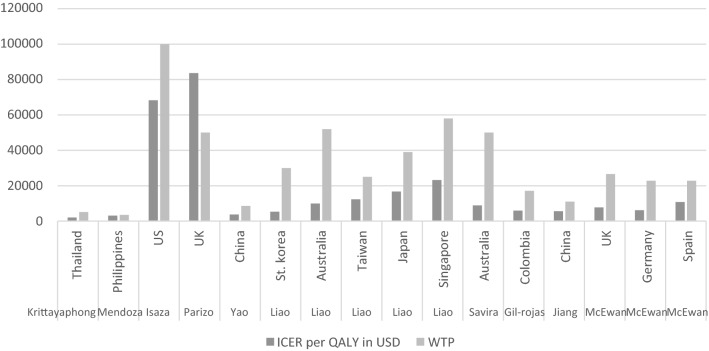
All ICERs reported in cost-effectiveness analyses per QALY gained

We converted all ICERs to US dollars for comparison across countries (Fig. 2). Two cost-utility analyses were performed in two different studies for the UK, Australia, and China. The highest and lowest WTPs were found in the US and the Philippines, and the highest and lowest ICERs were found in the US and Thailand, respectively. As can be seen in Fig. 2, the differences between ICERs vary widely in countries with different healthcare systems. However, in all cases, the ICER is below the WTP threshold and the new intervention is cost-effective. The only exception is Parizo et al. study, which was conducted in the UK NHS setting. The ICER of the dapagliflozin add-on arm was higher than the assumed WTP. According to the results, the only study that stated that dapagliflozin is not cost-effective at current prices was the study of Parizo et al. In this study, ICER fell below the WTP threshold by reducing drug prices from $450 to $270. [17].

Although dapagliflozin is cost-effective, its higher cost may increase the healthcare budget. However, for SGLT-2 inhibitors such as dapagliflozin, which are routinely prescribed for type 2 diabetes mellitus, the increased budget in practice would be less than the theoretically calculated budget [29–31]. Regarding the generalizability of the results of economic assessments, because studies have been conducted in both developed and developing countries, the distribution of the results is geographically and economically favorable. In general, the results of this systematic review can provide an economic perspective on the place of dapagliflozin in the world. However, the results of this study cannot be generalized to the whole world, and more economic evaluations, especially in developing countries, are needed in different contexts so that health policymakers in each country can evaluate cost-effectiveness and consequences under local data analysis. The results of this study are important for the policymakers because, despite a lot of research in the field of heart failure management, their prevalence and mortality rates are high, and the percentage of patients who are still alive more than 5 years after the diagnosis of heart failure it does not even reach 50% [2, 3]. Ivabradine and entresto^®^ (sacubitril + valsartan) can be mentioned among other drugs of great interest in heart failure. These drugs have also been the target of cost-effectiveness studies [32, 33]. However, one of the critical gaps in the economic evaluations related to heart failure is not comparing the cost-effectiveness of these drugs with dapagliflozin. Nevertheless, it is expected that due to the multi-indication of dapagliflozin compared to these two drugs, in other words, extensive benefits in diabetes and chronic kidney failure (CKD), the general interest of cardiologists in using dapagliflozin is more.

However, as with other similar studies, there were some limitations to this study that could affect the results. For one thing, real-world data were not recruited in the economic evaluations of most countries, and intra-individual variations were not accounted for in the different studies. Also, a number of economic assessments were published only in the form of reports, which were not included in the systematic review. Meta-analysis was not possible on the data obtained from the studies due to the high degree of heterogeneity and limited our study to a systematic review. For another, the current topic is one of the new topics in the scientific community, and clinical and economic research is dynamically updated, not only in diabetes and heart failure but also in some new topics such as CKD [11], so the results of similar studies in the future may affect the results of this review. One of the suggestions for future studies is the economic evaluation of dapagliflozin in CKD patients, which is one of the newest applications of SGLT-2i drugs.

## Conclusion

From the present systematic review results, it can be concluded that dapagliflozin, as a cardioprotective SGLT-2i, was cost-effective in most of the studied contexts and populations, especially in diabetic patients (See Additional file 1: Table S1).

## Supplementary Information

**Additional file 1: Table S1**. Search strategies and results in the databases and search engines.

## Data Availability

Not applicable.
