# Supplementary material for: Cost-effectiveness analysis of dapagliflozin in the management of heart failure with reduced ejection fraction (HFrEF): a systematic review
Source: Cost Eff Resour Alloc. 2022 Dec 1;20:62. doi: 10.1186/s12962-022-00396-7 (PMC9714165; doi:10.1186/s12962-022-00396-7)
Supplement: Supplementary file 1 — Additional file 1: Table S1. Search strategies and results in the databases and search engines. [file 12962_2022_396_MOESM1_ESM.docx]

| **Database** | **Search query** | **No** | **Filters applied** | **Final search result** |
| --- | --- | --- | --- | --- |
| **PubMed** | ("Dapagliflozin") AND ("cost-effectiveness" OR "cost-utility" OR "cost-benefit" OR "cost-minimization" OR "pharmacoeconomic study" OR "economic study" OR "pharmacoeconomic evaluation" OR "economic evaluation") AND ("heart failure") | 21 | *Research articles* |  |
| **Scopus** | ("Dapagliflozin") AND ("cost-effectiveness" OR "cost-utility" OR "cost-benefit" OR "cost-minimization" OR "pharmacoeconomic study" OR "economic study" OR "pharmacoeconomic evaluation" OR "economic evaluation") AND ("heart failure") | 42 | *Research articles* |  |
| **Web of Science** | ("Dapagliflozin") AND ("cost-effectiveness" OR "cost-utility" OR "cost-benefit" OR "cost-minimization" OR "pharmacoeconomic study" OR "economic study" OR "pharmacoeconomic evaluation" OR "economic evaluation") AND ("heart failure") | 20 | *Research articles* |  |
| **ScienceDirect** | ("Dapagliflozin") AND ("cost-effectiveness" OR "cost-utility" OR "cost-benefit" OR "cost-minimization" OR "pharmacoeconomic evaluation" OR "economic evaluation") AND ("heart failure") | 109 | *Research articles* | 1 |
| **Cochrane** | ("Dapagliflozin") AND ("cost-effectiveness" OR "cost-utility" OR "cost-benefit" OR "cost-minimization" OR "pharmacoeconomic study" OR "economic study" OR "pharmacoeconomic evaluation" OR "economic evaluation") AND ("heart failure") | 14 | *Research articles* |  |
